# Data Sharing Statements for Clinical Trials: A Requirement of the International Committee of Medical Journal Editors

**DOI:** 10.1371/journal.pmed.1002315

**Published:** 2017-06-05

**Authors:** Darren B. Taichman, Peush Sahni, Anja Pinborg, Larry Peiperl, Christine Laine, Astrid James, Sung-Tae Hong, Abraham Haileamlak, Laragh Gollogly, Fiona Godlee, Frank A. Frizelle, Fernando Florenzano, Jeffrey M. Drazen, Howard Bauchner, Christopher Baethge, Joyce Backus

**Affiliations:** 1 Secretary, ICMJE, Executive Deputy Editor, *Annals of Internal Medicine*; 2 Representative and Past President, World Association of Medical Editors; 3 Scientific Editor-in-Chief, *Ugeskrift for Laeger (Danish Medical Journal)*; 4 Chief Editor, *PLOS Medicine*; 5 Editor-in-Chief, *Annals of Internal Medicine*; 6 Deputy Editor, *The Lancet*; 7 Editor-in-Chief, *Journal of Korean Medical Science*; 8 Editor-in-Chief, *Ethiopian Journal of Health Sciences*; 9 Editor, *Bulletin of the World Health Organization*, Coordinator, WHO Press; 10 Editor-in-Chief, *The BMJ (British Medical Journal)*; 11 Editor-in-Chief, *New Zealand Medical Journal*; 12 Editor, *Revista Médica de Chile (Medical Journal of Chile)*; 13 Editor-in-Chief, *New England Journal of Medicine*; 14 Editor-in-Chief, *JAMA (Journal of the American Medical Association)* and the JAMA Network; 15 Chief Scientific Editor, *Deutsches Ärzteblatt (German Medical Journal) & Deutsches Ärzteblatt International*; 16 Representative and Associate Director for Library Operations, National Library of Medicine

## Abstract

The International Committee of Medical Journal Editors announces requirements that a data sharing plan be prospectively registered, and a data sharing statement be included in submitted manuscripts, for clinical trials to be published in ICMJE journals.

The International Committee of Medical Journal Editors (ICMJE) believes there is an ethical obligation to responsibly share data generated by interventional clinical trials because trial participants have put themselves at risk. In January 2016 we published a proposal aimed at helping to create an environment in which the sharing of deidentified individual participant data becomes the norm. In response to our request for feedback we received many comments from individuals and groups [1]. Some applauded the proposals while others expressed disappointment they did not more quickly create a commitment to data sharing. Many raised valid concerns regarding the feasibility of the proposed requirements, the necessary resources, the real or perceived risks to trial participants, and the need to protect the interests of patients and researchers.

It is encouraging that data sharing is already occurring in some settings. Over the past year, however, we have learned that the challenges are substantial and the requisite mechanisms are not in place to mandate universal data sharing at this time. Although many issues must be addressed for data sharing to become the norm, we remain committed to this goal.

Therefore, ICMJE will require the following as conditions of consideration for publication of a clinical trial report in our member journals:

1. As of July 1, 2018 manuscripts submitted to ICMJE journals that report the results of clinical trials must contain a data sharing statement as described below.
2. Clinical trials that begin enrolling participants on or after January 1, 2019 must include a data sharing plan in the trial’s registration. The ICMJE’s policy regarding trial registration is explained at *www.icmje.org/recommendations/browse/publishing-and-editorial-issues/clinical-trial-registration.html*. If the data sharing plan changes after registration this should be reflected in the statement submitted and published with the manuscript, and updated in the registry record.

Data sharing statements must indicate the following: whether individual deidentified participant data (including data dictionaries) will be shared; what data in particular will be shared; whether additional, related documents will be available (e.g., study protocol, statistical analysis plan, etc.); when the data will become available and for how long; by what access criteria data will be shared (including with whom, for what types of analyses and by what mechanism). Illustrative examples of data sharing statements that would meet these requirements are in Table 1.

**Table 1:** Examples of data sharing statements that fulfill these ICMJE requirements*.

	Example 1	Example 2	Example 3	Example 4
**Will individual participant data be available (including data dictionaries)?**	Yes	Yes	Yes	No
**What data in particular will be shared?**	All of the individual participant data collected during the trial, after deidentification.	Individual participant data that underlie the results reported in this article, after deidentification (text, tables, figures, and appendices).	Individual participant data that underlie the results reported in this article, after deidentification (text, tables, figures and appendices).	Not available
**What other documents will be available?**	Study Protocol, Statistical Analysis Plan, Informed Consent Form, Clinical Study Report, Analytic Code	Study Protocol, Statistical Analysis Plan, Analytic Code	Study Protocol	Not available
**When will data be available (start and end dates)?**	Immediately following publication. No end date.	Beginning 3 months and ending 5 years following article publication.	Beginning 9 months and ending 36 months following article publication.	Not applicable
**With whom?**	Anyone who wishes to access the data.	Researchers who provide a methodologically sound proposal.	Investigators whose proposed use of the data has been approved by an independent review committee (“learned intermediary”) identified for this purpose.	Not applicable
**For what types of analyses?**	Any purpose.	To achieve aims in the approved proposal.	For individual participant data meta-analysis.	Not applicable
**By what mechanism will data be made available?**	Data are available indefinitely at (*Link to be included)*.	Proposals should be directed to xxx@yyy. To gain access, data requestors will need to sign a data access agreement. Data are available for 5 years at a third party website. (*Link to be included)*	Proposals may be submitted up to 36 months following article publication. After 36 months the data will be available in our University’s data warehouse but without investigator support other than deposited metadata. Information regarding submitting proposals and accessing data may be found at (*Link to be provided*).	Not applicable

*These examples are meant to illustrate a range of, but not all, data sharing options.

These initial requirements do not yet mandate data sharing, but investigators should be aware that editors may take into consideration data sharing statements when making editorial decisions. These minimum requirements are intended to move the research enterprise closer to fulfilling our ethical obligation to participants. Some ICMJE member journals already maintain, or may choose to adopt, more stringent requirements for data sharing.

Sharing clinical trial data is one step in the process articulated by the World Health Organization (WHO) and other professional organizations as best practice for clinical trials: universal prospective registration; public disclosure of results from all clinical trials (including through journal publication); and data sharing. Although universal compliance with the requirement to prospectively register clinical trials has not yet been achieved and requires continued emphasis, we must work toward fulfilling the other steps of best practice as well–including data sharing.

As we move forward into this new norm where data are shared, greater understanding and collaboration among funders, ethics committees, journals, trialists, data analysts, participants, and others will be required. We are currently working with members of the research community to facilitate practical solutions to enable data sharing. The United States Office for Human Research Protections has indicated that provided the appropriate conditions are met by those receiving them, the sharing of deidentified individual participant data from clinical trials does not require separate consent from trial participants [2]. Specific elements to enable data sharing statements that meet these requirements have been adopted at ClinicalTrials.gov (https://prsinfo.clinicaltrials.gov/definitions.html#shareData). The WHO also supports the addition of such elements at the primary registries of the International Clinical Trials Registry Platform. Unresolved issues remain, including appropriate scholarly credit to those who share data, and the resources needed for data access, the transparent processing of data requests, and data archiving. We welcome creative solutions to these problems at www.icmje.org.

We envision a global research community in which sharing deidentified data becomes the norm. Working toward this vision will help maximize the knowledge gained from the efforts and sacrifices of clinical trial participants.
